# Mathematics of microRNAs: stabilizing gene regulatory networks

**DOI:** 10.1093/nsr/nwz112

**Published:** 2019-08-13

**Authors:** Victor Ambros

**Affiliations:** Program in Molecular Medicine, University of Massachusetts Medical School, UK

MicroRNAs (miRNAs) are fascinating in their versatility. Animal cells express hundreds of distinct microRNAs, and each microRNA can regulate hundreds of distinct mRNA targets. Therefore, in aggregate, the microRNA compliment of a cell can exert pervasive effects on gene expression. Individual microRNAs can also be potent determinants of phenotype: The first microRNAs identified by genetics in nematodes [1,2] and Drosophila [3] function as master regulators controlling genetic switches between cell fates, largely by repressing translation of the mRNA of a single target—for example, *lin-41* in the case of the *let-7* microRNA [4], or Hid in the case of *bantam* [3].

Many additional cases of microRNAs functioning as developmental switches have been identified genetically, but also many puzzling cases have emerged where genetic ablation of a microRNA—or even entire families of microRNAs—resulted in no visible phenotype [5,6]. In some such cases the microRNA turns out to be required under certain stressful conditions to ensure robust development [7–9]. These findings led to the appreciation of the importance of microRNAs for buffering genetic regulatory pathways against the perturbations associated with everyday stresses, such as temperature fluctuations [7,8], or pathogen challenge [9].

The fact that microRNAs are predicted to target thousands of genes—even a ‘developmental switch’ microRNA such as *let-7* has hundreds of complementary mRNA targets in addition to *lin-41*—has suggested that a major role for microRNAs could be in conferring developmental or physiological robustness, essentially by dampening transcriptional noise or other fluctuations in gene regulatory networks (GRNs) [10]. Mechanistically, this buffering function has been considered to reflect a default mode of microRNA activity, where microRNA binding to target mRNA causes a slight destabilization of the mRNA, without necessarily inhibition of protein synthesis [11]. However, it has also been shown that in vivo, microRNAs can engage in a dynamic process of repression, with translational inhibition preceding target mRNA destabilization [12].

So, the versatile microRNA can in some cases directly affect specific phenotypes, often by translational repression of a limited set of mRNA targets, or alternatively (or even simultaneously), that same microRNA can pitch in with hundreds of other microRNAs to collectively dampen gene expression across the transcriptome.

Given the assumption that the majority of microRNA::target interactions could be weak, and individually exert only slight reduction of gene activity, can this pervasive pan-transcriptome activity of microRNAs contribute to the stability of the gene regulatory networks that underlie cellular physiology and behavior? Chung-I Wu and co-workers (Chen *et al*.) explore this question using mathematical modelling of GRN stability against perturbations, employing network parameters that are anchored by actual transcriptomic data, together with data-guided assumptions for the genomic scope and potency of microRNA repression. By modelling GRN stability with or without varying degrees of microRNA activity, Chen *et al*. show that even weak microRNA::target interactions, when applied across a substantial fraction of the transcriptome, are sufficient to exert stabilizing effects on the network [13].

The results of the Chen *et al*. study reinforce our appreciation of the versatility of microRNAs—with their capacity to function, on the one hand, as master regulators of phenotypically-potent developmental switches, and on the other hand, as minor contributors to a powerful pan-transcriptomic stabilizing collective.

Future directions for this sort of modelling of GRN stability could include employing ribosome profiling datasets to incorporate into the model the impact of microRNAs on the proteome, represented by mRNA translational activity. Since microRNAs can exert translational repression that is often more potent than their effects on mRNA levels [3,14], the network stabilizing effects of microRNAs could likewise be more potent when assessed in terms of protein synthesis.

It would also be interesting to use the Chen *et al* approach to explore the contribution of microRNAs to GRN stability in the face of specific biological stresses such as heat shock response, inflammation, or cell cycle arrest/progression, with the perturbation models based on datasets of gene expression dynamics during those processes.

## References

[1] Lee RC , Feinbaum RL, Ambros V. Cell 1993; 75: 843–54. 8252621 10.1016/0092-8674(93)90529-y

[2] Reinhart BJ , Slack FJ, Basson M et al. Nature 2000; 403: 901–6. 10706289 10.1038/35002607

[3] Brennecke J , Hipfner DR, Stark A et al. Cell 2003; 113: 25–36. 12679032 10.1016/s0092-8674(03)00231-9

[4] Slack FJ , Basson M, Liu Z et al. Mol Cell 2000; 5: 659–69. 10882102 10.1016/s1097-2765(00)80245-2

[5] Alvarez-Saavedra E , Horvitz HR. Curr Biol 2010; 20: 367–73. 20096582 10.1016/j.cub.2009.12.051 PMC2844791

[6] Miska EA , Alvarez-Saavedra E, Abbott AL et al. PLoS Genet 2007; 3: e215. 18085825 10.1371/journal.pgen.0030215 PMC2134938

[7] Burke SL , Hammell M, Ambros V. Genetics 2015; 200: 1201–18. 26078280 10.1534/genetics.115.179184 PMC4574240

[8] Li X , Cassidy JJ, Reinke CA et al. Cell 2009; 137: 273–82. 19379693 10.1016/j.cell.2009.01.058 PMC2674871

[9] Ren Z , Ambros VR. Proc Natl Acad Sci USA 2015; 112: E2366–75. 25897023 10.1073/pnas.1422858112 PMC4426397

[10] Ebert MS , Sharp PA. Cell 2012; 149: 515–24. 22541426 10.1016/j.cell.2012.04.005 PMC3351105

[11] Eichhorn SW , Guo H, McGeary SE et al. Mol Cell 2014; 56: 104–15. 25263593 10.1016/j.molcel.2014.08.028 PMC4292926

[12] Bazzini AA , Lee MT, Giraldez AJ. Science 2012; 336: 233–7. 22422859 10.1126/science.1215704 PMC3547538

[13] Chen YX , Shen Y, Lin P et al. Natl Sci Rev 2019; 6: 1176–88. 10.1093/nsr/nwz076 PMC8291590 34691996

[14] Stadler M , Artiles K, Pak J et al. Genome Res 2012; 22: 2418–26. 22855835 10.1101/gr.136515.111 PMC3514671

